# Supplementary material for: A Likelihood Approach for Real-Time Calibration of Stochastic Compartmental Epidemic Models
Source: PLoS Comput Biol. 2017 Jan 17;13(1):e1005257. doi: 10.1371/journal.pcbi.1005257 (PMC5240920; doi:10.1371/journal.pcbi.1005257)
Supplement: S1 File — (TAR.GZ) [file pcbi.1005257.s014.tar.gz › HSPH_Online-SI-Revision/MSS10/n100-extreme/n100-extreme_NEW2_table8.pdf]

| | | | | | | | | | | | | | | | | | | | | | | | | | | | | | | |
|---|---|---|---|---|---|---|---|---|---|---|---|---|---|---|---|---|---|---|---|---|---|---|---|---|---|---|---|---|---|---|
| 1 | newly 3 weeks spec | 655. | 602.86 | {568., 630.} | 8.05191 | 0.0367304 | newly 3 weeks cumul | 2650. | 2519.01 | {2479., 2566.} | 4.94302 | 0.0220502 | newly 5 weeks spec | 298. | 323.94 | {299., 345.} | 8.95302 | 0.0367265 | newly 5 weeks cumul | 3391. | 3283.89 | {3234., 3325.} | 3.15866 | 0.0139631 | newly 46 weeks cumul | 4195. | 4171.26 | {4110., 4232.} | 1.02169 | 0.00445906 |
| 2 | newly 3 weeks spec | 761. | 763.47 | {730., 804.} | 3.02365 | 0.0130489 | newly 3 weeks cumul | 3077. | 3033.29 | {2983., 3081.} | 1.56939 | 0.00689394 | newly 5 weeks spec | 441. | 441.17 | {415., 466.} | 3.5034 | 0.0152416 | newly 5 weeks cumul | 4068. | 4058.17 | {3986., 4115.} | 0.959931 | 0.00418129 | newly 42 weeks cumul | 5247. | 5477.1 | {5376., 5580.} | 4.39451 | 0.018633 |
| 3 | newly 3 weeks spec | 277. | 264.28 | {242., 283.} | 5.89892 | 0.0267475 | newly 3 weeks cumul | 1321. | 1234.6 | {1202., 1266.} | 6.5405 | 0.0294523 | newly 5 weeks spec | 122. | 117.29 | {104., 131.} | 8.09016 | 0.0364088 | newly 5 weeks cumul | 1607. | 1528.03 | {1487., 1558.} | 4.91413 | 0.0219481 | newly 47 weeks cumul | 1853. | 1760.17 | {1718., 1807.} | 5.04965 | 0.0225893 |
| 4 | newly 3 weeks spec | 75. | 52.84 | {45., 62.} | 29.5467 | 0.156192 | newly 3 weeks cumul | 495. | 311.88 | {295., 328.} | 36.9939 | 0.201029 | newly 5 weeks spec | 26. | 18.51 | {12., 25.} | 29.6538 | 0.170481 | newly 5 weeks cumul | 564. | 361.72 | {341., 381.} | 35.8652 | 0.193323 | newly 51 weeks cumul | 589. | 385.93 | {364., 405.} | 34.4771 | 0.184044 |
| 5 | newly 3 weeks spec | 383. | 306.17 | {285., 329.} | 20.0601 | 0.0980066 | newly 3 weeks cumul | 1884. | 1444.51 | {1408., 1473.} | 23.3275 | 0.115426 | newly 5 weeks spec | 164. | 134.83 | {120., 151.} | 17.8232 | 0.0870659 | newly 5 weeks cumul | 2324. | 1783.52 | {1748., 1818.} | 23.2565 | 0.115015 | newly 48 weeks cumul | 2679. | 2052.93 | {1996., 2108.} | 23.3695 | 0.115689 |
| 6 | newly 3 weeks spec | 620. | 606.13 | {571., 637.} | 3.71129 | 0.0164571 | newly 3 weeks cumul | 2591. | 2675.05 | {2629., 2718.} | 3.24701 | 0.01384 | newly 5 weeks spec | 303. | 300.12 | {273., 322.} | 4.61386 | 0.0202753 | newly 5 weeks cumul | 3314. | 3402.77 | {3347., 3451.} | 2.67864 | 0.0114524 | newly 46 weeks cumul | 4005. | 4112.58 | {4050., 4172.} | 2.68964 | 0.0114974 |
| 7 | newly 3 weeks spec | 425. | 744.08 | {714., 776.} | 75.0776 | 0.242991 | newly 3 weeks cumul | 2093. | 3082.62 | {3033., 3127.} | 47.2824 | 0.16812 | newly 5 weeks spec | 238. | 407.02 | {377., 433.} | 71.0168 | 0.232466 | newly 5 weeks cumul | 2658. | 4043.13 | {3984., 4095.} | 52.1117 | 0.182139 | newly 44 weeks cumul | 3121. | 5188.53 | {5129., 5254.} | 66.2458 | 0.220731 |
| 8 | newly 3 weeks spec | 611. | 721.69 | {686., 752.} | 18.1162 | 0.0720215 | newly 3 weeks cumul | 2634. | 3092.64 | {3039., 3139.} | 17.4123 | 0.0696854 | newly 5 weeks spec | 315. | 373.42 | {348., 402.} | 18.546 | 0.0732256 | newly 5 weeks cumul | 3371. | 3988.35 | {3945., 4039.} | 18.3136 | 0.0730167 | newly 46 weeks cumul | 4133. | 4952.29 | {4891., 4999.} | 19.8231 | 0.0785239 |
| 9 | newly 3 weeks spec | 509. | 477.73 | {445., 507.} | 6.39882 | 0.029105 | newly 3 weeks cumul | 2442. | 2259.51 | {2226., 2297.} | 7.47297 | 0.0337661 | newly 5 weeks spec | 256. | 210.76 | {191., 228.} | 17.6719 | 0.0855172 | newly 5 weeks cumul | 3053. | 2791.39 | {2744., 2834.} | 8.56895 | 0.0389362 | newly 46 weeks cumul | 3616. | 3211.73 | {3159., 3274.} | 11.18 | 0.0515309 |
| 10 | newly 3 weeks spec | 32. | 16.26 | {8., 27.} | 50.3125 | 0.350351 | newly 3 weeks cumul | 165. | 108.45 | {79., 139.} | 34.3091 | 0.191501 | newly 5 weeks spec | 7. | 4.73 | {0., 10.} | 56.4286 | 0.514093 | newly 5 weeks cumul | 184. | 122.01 | {88., 160.} | 34.0815 | 0.190584 | newly 50 weeks cumul | 192. | 127.5 | {89., 170.} | 34.2917 | 0.192911 |
| 11 | newly 3 weeks spec | 582. | 924.63 | {882., 958.} | 58.8711 | 0.200811 | newly 3 weeks cumul | 2565. | 3936.43 | {3881., 3983.} | 53.4671 | 0.185995 | newly 5 weeks spec | 318. | 483.8 | {451., 518.} | 52.1384 | 0.181704 | newly 5 weeks cumul | 3307. | 5084.61 | {5023., 5142.} | 53.7529 | 0.186806 | newly 44 weeks cumul | 4044. | 6324.49 | {6229., 6408.} | 56.3919 | 0.194185 |
| 12 | newly 3 weeks spec | 488. | 401.42 | {376., 425.} | 17.7418 | 0.0852834 | newly 3 weeks cumul | 2113. | 1793.04 | {1754., 1826.} | 15.1425 | 0.0713612 | newly 5 weeks spec | 223. | 197.45 | {181., 214.} | 11.6278 | 0.0545504 | newly 5 weeks cumul | 2630. | 2273.66 | {2235., 2308.} | 13.549 | 0.0632663 | newly 45 weeks cumul | 3083. | 2736.1 | {2690., 2776.} | 11.252 | 0.0518723 |
| 13 | newly 3 weeks spec | 1177. | 757.48 | {721., 788.} | 35.6432 | 0.191706 | newly 3 weeks cumul | 4644. | 3355.77 | {3305., 3404.} | 27.7397 | 0.141133 | newly 5 weeks spec | 679. | 370.41 | {342., 395.} | 45.4477 | 0.263922 | newly 5 weeks cumul | 6187. | 4258.87 | {4199., 4322.} | 31.1642 | 0.162216 | newly 43 weeks cumul | 8302. | 5123.35 | {5025., 5204.} | 38.2878 | 0.209675 |
| 14 | newly 3 weeks spec | 9. | 7.87 | {1., 17.} | 58.3333 | 0.407398 | newly 3 weeks cumul | 64. | 71.43 | {38., 107.} | 31.4531 | 0.132837 | newly 5 weeks spec | 5. | 1.79 | {0., 5.} | 79. | 1.05266 | newly 5 weeks cumul | 74. | 76.77 | {41., 123.} | 30.0676 | 0.137389 | newly 48 weeks cumul | 86. | 77.61 | {41., 127.} | 28.0581 | 0.145933 |
| 15 | newly 3 weeks spec | 73. | 1.79 | {0., 5.} | 97.5479 | 1.98927 | newly 3 weeks cumul | 382. | 10.81 | {3., 20.} | 97.1702 | 1.66787 | newly 5 weeks spec | 19. | 0.5 | {0., 2.} | 97.3684 | 1.94531 | newly 5 weeks cumul | 449. | 12.47 | {3., 24.} | 97.2227 | 1.68492 | newly 48 weeks cumul | 476. | 13.06 | {3., 26.} | 97.2563 | 1.69715 |
| 16 | newly 3 weeks spec | 111. | 124.16 | {110., 137.} | 13.009 | 0.0519239 | newly 3 weeks cumul | 653. | 669.06 | {649., 690.} | 2.93109 | 0.012496 | newly 5 weeks spec | 31. | 45.64 | {37., 56.} | 47.2258 | 0.161964 | newly 5 weeks cumul | 748. | 789.64 | {763., 818.} | 5.62032 | 0.0236031 | newly 48 weeks cumul | 812. | 858.45 | {823., 890.} | 5.81897 | 0.0243854 |
| 17 | newly 3 weeks spec | 848. | 642.71 | {612., 675.} | 24.2087 | 0.120661 | newly 3 weeks cumul | 3379. | 2650.01 | {2605., 2695.} | 21.5741 | 0.105577 | newly 5 weeks spec | 459. | 353.41 | {328., 381.} | 23.0044 | 0.114191 | newly 5 weeks cumul | 4480. | 3483.88 | {3428., 3535.} | 22.2348 | 0.109246 | newly 43 weeks cumul | 5685. | 4483.07 | {4404., 4572.} | 21.1421 | 0.103197 |
| 18 | newly 3 weeks spec | 371. | 608.08 | {570., 643.} | 63.903 | 0.214112 | newly 3 weeks cumul | 1789. | 2480.92 | {2432., 2532.} | 38.6764 | 0.141944 | newly 5 weeks spec | 168. | 341.36 | {314., 365.} | 103.19 | 0.307068 | newly 5 weeks cumul | 2207. | 3275.8 | {3221., 3341.} | 48.4277 | 0.171469 | newly 44 weeks cumul | 2601. | 4257.41 | {4142., 4363.} | 63.6836 | 0.213918 |
| 19 | newly 3 weeks spec | 522. | 716.99 | {685., 747.} | 37.3544 | 0.13759 | newly 3 weeks cumul | 2300. | 3238.4 | {3193., 3283.} | 40.8 | 0.148577 | newly 5 weeks spec | 225. | 342.48 | {320., 362.} | 52.2133 | 0.18184 | newly 5 weeks cumul | 2858. | 4076.53 | {4037., 4115.} | 42.6358 | 0.154215 | newly 46 weeks cumul | 3344. | 4836.79 | {4783., 4886.} | 44.6408 | 0.160279 |
| 20 | newly 3 weeks spec | 31. | 93.07 | {78., 110.} | 200.226 | 0.473573 | newly 3 weeks cumul | 168. | 723.32 | {695., 751.} | 330.548 | 0.633855 | newly 5 weeks spec | 4. | 19.64 | {13., 26.} | 391. | 0.676088 | newly 5 weeks cumul | 185. | 786.47 | {753., 816.} | 325.119 | 0.628322 | newly 51 weeks cumul | 187. | 803.57 | {767., 835.} | 329.717 | 0.632994 |
| 21 | newly 3 weeks spec | 203. | 196.22 | {177., 216.} | 5.99015 | 0.0268701 | newly 3 weeks cumul | 1027. | 914.7 | {884., 946.} | 10.9348 | 0.0504474 | newly 5 weeks spec | 76. | 89.09 | {70., 104.} | 21.0658 | 0.0823376 | newly 5 weeks cumul | 1233. | 1137. | {1086., 1182.} | 7.78589 | 0.0353864 | newly 48 weeks cumul | 1341. | 1319.74 | {1258., 1375.} | 3.10365 | 0.0136842 |
| 22 | newly 3 weeks spec | 138. | 489.32 | {460., 518.} | 254.58 | 0.54923 | newly 3 weeks cumul | 788. | 2542.81 | {2494., 2594.} | 222.692 | 0.508738 | newly 5 weeks spec | 76. | 183.98 | {162., 205.} | 142.079 | 0.382175 | newly 5 weeks cumul | 953. | 3028.6 | {2970., 3092.} | 217.796 | 0.502097 | newly 49 weeks cumul | 1046. | 3324.46 | {3238., 3418.} | 217.826 | 0.502104 |
| 23 | newly 3 weeks spec | 646. | 666.45 | {636., 694.} | 4.07585 | 0.0172846 | newly 3 weeks cumul | 2717. | 2659.27 | {2609., 2702.} | 2.22635 | 0.00980613 | newly 5 weeks spec | 343. | 389.3 | {364., 415.} | 13.5277 | 0.0545525 | newly 5 weeks cumul | 3563. | 3563.84 | {3504., 3611.} | 0.961549 | 0.00417798 | newly 42 weeks cumul | 4442. | 4830.61 | {4743., 4905.} | 8.74854 | 0.0363814 |
| 24 | newly 3 weeks spec | 888. | 1285.16 | {1238., 1328.} | 44.7252 | 0.160377 | newly 3 weeks cumul | 3507. | 5409.4 | {5349., 5469.} | 54.2458 | 0.188196 | newly 5 weeks spec | 441. | 684.42 | {649., 716.} | 55.1973 | 0.190612 | newly 5 weeks cumul | 4589. | 7034.03 | {6975., 7094.} | 53.2802 | 0.185478 | newly 42 weeks cumul | 5895. | 8890.13 | {8844., 8936.} | 50.808 | 0.178421 |
| 25 | newly 3 weeks spec | 135. | 175.43 | {158., 194.} | 29.9481 | 0.112315 | newly 3 weeks cumul | 801. | 1031.68 | {1003., 1062.} | 28.799 | 0.109807 | newly 5 weeks spec | 46. | 56.47 | {47., 67.} | 23.5 | 0.0881667 | newly 5 weeks cumul | 936. | 1185.89 | {1147., 1226.} | 26.6976 | 0.10264 | newly 45 weeks cumul | 1018. | 1260.34 | {1212., 1303.} | 23.8055 | 0.0925804 |
| 26 | newly 3 weeks spec | 1048. | 466.32 | {443., 493.} | 55.5038 | 0.352131 | newly 3 weeks cumul | 4173. | 2065.91 | {2019., 2104.} | 50.4934 | 0.305394 | newly 5 weeks spec | 644. | 230.44 | {211., 250.} | 64.2174 | 0.447288 | newly 5 weeks cumul | 5651. | 2625.86 | {2571., 2670.} | 53.5328 | 0.332898 | newly 43 weeks cumul | 7533. | 3176.4 | {3102., 3243.} | 57.8335 | 0.375101 |
| 27 | newly 3 weeks spec | 806. | 1026.38 | {991., 1080.} | 27.3424 | 0.104759 | newly 3 weeks cumul | 3332. | 4230.37 | {4177., 4278.} | 26.9619 | 0.103652 | newly 5 weeks spec | 451. | 563.33 | {528., 598.} | 24.9069 | 0.0961527 | newly 5 weeks cumul | 4401. | 5556.02 | {5508., 5603.} | 26.2445 | 0.101202 | newly 44 weeks cumul | 5811. | 7179.09 | {7121., 7238.} | 23.5431 | 0.09181 |
| 28 | newly 3 weeks spec | 568. | 302.64 | {279., 325.} | 46.7183 | 0.274157 | newly 3 weeks cumul | 2482. | 1438.02 | {1405., 1469.} | 42.062 | 0.237104 | newly 5 weeks spec | 266. | 132.3 | {118., 148.} | 50.2632 | 0.305145 | newly 5 weeks cumul | 3132. | 1770.87 | {1733., 1806.} | 43.4588 | 0.247686 | newly 48 weeks cumul | 3853. | 2037.14 | {1991., 2083.} | 47.1285 | 0.276836 |
| 29 | newly 3 weeks spec | 769. | 504.18 | {468., 542.} | 34.4369 | 0.184043 | newly 3 weeks cumul | 3241. | 2173.86 | {2119., 2232.} | 32.9263 | 0.173525 | newly 5 weeks spec | 412. | 260.62 | {237., 286.} | 36.7427 | 0.200125 | newly 5 weeks cumul | 4213. | 2796.56 | {2741., 2851.} | 33.6207 | 0.178029 | newly 42 weeks cumul | 5339. | 3440.9 | {3340., 3531.} | 35.5516 | 0.1909 |
| 30 | newly 3 weeks spec | 278. | 238.87 | {215., 261.} | 14.0755 | 0.0670013 | newly 3 weeks cumul | 1314. | 1182.08 | {1147., 1216.} | 10.0396 | 0.0460376 | newly 5 weeks spec | 87. | 98.22 | {82., 112.} | 15.1034 | 0.0600295 | newly 5 weeks cumul | 1559. | 1434.01 | {1398., 1466.} | 8.01732 | 0.0363717 | newly 51 weeks cumul | 1722. | 1606.54 | {1562., 1644.} | 6.70499 | 0.0302225 |
| 31 | newly 3 weeks spec | 58. | 185.94 | {165., 203.} | 220.586 | 0.504421 | newly 3 weeks cumul | 360. | 1049.2 | {1019., 1077.} | 191.444 | 0.464449 | newly 5 weeks spec | 22. | 63.54 | {52., 75.} | 188.818 | 0.455994 | newly 5 weeks cumul | 413. | 1220.28 | {1184., 1257.} | 195.467 | 0.470376 | newly 52 weeks cumul | 442. | 1308.14 | {1266., 1354.} | 195.959 | 0.471077 |
| 32 | newly 3 weeks spec | 101. | 355.47 | {329., 384.} | 251.95 | 0.545692 | newly 3 weeks cumul | 549. | 1759.56 | {1701., 1807.} | 220.503 | 0.505719 | newly 5 weeks spec | 30. | 149.55 | {128., 170.} | 398.5 | 0.694932 | newly 5 weeks cumul | 633. | 2135.35 | {2075., 2194.} | 237.338 | 0.527937 | newly 49 weeks cumul | 682. | 2404.16 | {2318., 2505.} | 252.516 | 0.546963 |
| 33 | newly 3 weeks spec | 918. | 643.95 | {610., 674.} | 29.8529 | 0.154319 | newly 3 weeks cumul | 3832. | 2719.29 | {2678., 2761.} | 29.0373 | 0.149004 | newly 5 weeks spec | 479. | 343.79 | {319., 367.} | 28.2276 | 0.144846 | newly 5 weeks cumul | 4959. | 3536.38 | {3493., 3579.} | 28.6876 | 0.146855 | newly 47 weeks cumul | 6261. | 4463.65 | {4380., 4536.} | 28.7071 | 0.14699 |
| 34 | newly 3 weeks spec | 843. | 1287.06 | {1233., 1332.} | 52.6762 | 0.1836 | newly 3 weeks cumul | 3518. | 4852.81 | {4785., 4915.} | 37.9423 | 0.13967 | newly 5 weeks spec | 471. | 820.17 | {776., 859.} | 74.1338 | 0.240588 | newly 5 weeks cumul | 4638. | 6702.39 | {6593., 6801.} | 44.5103 | 0.159872 | newly 42 weeks cumul | 6028. | 9828.18 | {9662., 9981.} | 63.0421 | 0.212264 |
| 35 | newly 3 weeks spec | 693. | 813.36 | {778., 847.} | 17.368 | 0.0693253 | newly 3 weeks cumul | 3121. | 3662.28 | {3620., 3707.} | 17.3432 | 0.0694377 | newly 5 weeks spec | 338. | 391.17 | {362., 420.} | 15.7367 | 0.062834 | newly 5 weeks cumul | 3977. | 4616.85 | {4572., 4656.} | 16.0888 | 0.0647792 | newly 47 weeks cumul | 4760. | 5493.62 | {5437., 5549.} | 15.4122 | 0.0622343 |
| 36 | newly 3 weeks spec | 635. | 1136.19 | {1095., 1174.} | 78.9276 | 0.25251 | newly 3 weeks cumul | 2904. | 4722.95 | {4667., 4777.} | 62.636 | 0.211198 | newly 5 weeks spec | 364. | 623.27 | {589., 657.} | 71.228 | 0.23315 | newly 5 weeks cumul | 3816. | 6194.57 | {6136., 6259.} | 62.3315 | 0.210389 | newly 45 weeks cumul | 4844. | 7927.66 | {7802., 8032.} | 63.6594 | 0.213913 |
| 37 | newly 3 weeks spec | 121. | 34.85 | {27., 44.} | 71.1983 | 0.548384 | newly 3 weeks cumul | 634. | 194.23 | {171., 214.} | 69.3644 | 0.515198 | newly 5 weeks spec | 49. | 12.5 | {7., 18.} | 74.4898 | 0.622425 | newly 5 weeks cumul | 759. | 227.26 | {197., 250.} | 70.058 | 0.525244 | newly 50 weeks cumul | 824. | 243.9 | {212., 273.} | 70.4005 | 0.53037 |
| 38 | newly 3 weeks spec | 12. | 5.67 | {2., 10.} | 56.4167 | 0.465133 | newly 3 weeks cumul | 113. | 42.61 | {25., 57.} | 62.292 | 0.441919 | newly 5 weeks spec | 3. | 1.46 | {0., 3.} | 66. | 0.729303 | newly 5 weeks cumul | 121. | 46.49 | {28., 66.} | 61.5785 | 0.435219 | newly 50 weeks cumul | 126. | 47.72 | {28., 68.} | 62.127 | 0.442931 |
| 39 | newly 3 weeks spec | 302. | 179.17 | {151., 202.} | 40.6722 | 0.228956 | newly 3 weeks cumul | 1471. | 801.57 | {743., 849.} | 45.5085 | 0.264297 | newly 5 weeks spec | 115. | 86.82 | {69., 104.} | 24.5043 | 0.127178 | newly 5 weeks cumul | 1796. | 1012.65 | {936., 1082.} | 43.6164 | 0.249556 | newly 44 weeks cumul | 2002. | 1212.32 | {1095., 1325.} | 39.4446 | 0.219029 |
| 40 | newly 3 weeks spec | 529. | 837.68 | {794., 882.} | 58.3516 | 0.19932 | newly 3 weeks cumul | 2325. | 3766.74 | {3721., 3812.} | 62.0103 | 0.209523 | newly 5 weeks spec | 275. | 400.86 | {369., 427.} | 45.7673 | 0.162996 | newly 5 weeks cumul | 2963. | 4746.33 | {4674., 4801.} | 60.1866 | 0.204601 | newly 45 weeks cumul | 3697. | 5635.28 | {5549., 5714.} | 52.4285 | 0.183036 |
| 41 | newly 3 weeks spec | 253. | 160.73 | {139., 185.} | 36.4704 | 0.199632 | newly 3 weeks cumul | 1228. | 829.73 | {769., 879.} | 32.4324 | 0.170863 | newly 5 weeks spec | 114. | 61.25 | {48., 75.} | 46.2719 | 0.276651 | newly 5 weeks cumul | 1511. | 990.64 | {920., 1056.} | 34.4381 | 0.183997 | newly 46 weeks cumul | 1685. | 1095.07 | {988., 1186.} | 35.0107 | 0.188084 |
| 42 | newly 3 weeks spec | 23. | 61.79 | {43., 81.} | 168.652 | 0.416978 | newly 3 weeks cumul | 166. | 406.21 | {355., 457.} | 144.705 | 0.386716 | newly 5 weeks spec | 8. | 17.94 | {9., 30.} | 127.75 | 0.327356 | newly 5 weeks cumul | 186. | 457. | {394., 524.} | 145.699 | 0.387931 | newly 50 weeks cumul | 187. | 476.65 | {398., 551.} | 154.893 | 0.403539 |
| 43 | newly 3 weeks spec | 681. | 1071.09 | {1028., 1110.} | 57.2819 | 0.196508 | newly 3 weeks cumul | 3118. | 4410.76 | {4355., 4458.} | 41.4612 | 0.150621 | newly 5 weeks spec | 377. | 588.04 | {558., 615.} | 55.9788 | 0.192736 | newly 5 weeks cumul | 4043. | 5789.48 | {5737., 5843.} | 43.1976 | 0.155925 | newly 43 weeks cumul | 5018. | 7465.67 | {7418., 7514.} | 48.7778 | 0.172532 |
| 44 | newly 3 weeks spec | 174. | 270.85 | {249., 295.} | 55.6609 | 0.191252 | newly 3 weeks cumul | 824. | 1364.3 | {1329., 1395.} | 65.5704 | 0.218911 | newly 5 weeks spec | 61. | 105.58 | {91., 120.} | 73.082 | 0.235494 | newly 5 weeks cumul | 988. | 1638.46 | {1607., 1672.} | 65.836 | 0.219623 | newly 49 weeks cumul | 1084. | 1822.25 | {1783., 1858.} | 68.1042 | 0.22551 |
| 45 | newly 3 weeks spec | 45. | 78.37 | {63., 90.} | 74.1556 | 0.236693 | newly 3 weeks cumul | 244. | 542.29 | {511., 574.} | 122.25 | 0.346343 | newly 5 weeks spec | 9. | 20.93 | {14., 28.} | 133.222 | 0.351659 | newly 5 weeks cumul | 276. | 603.2 | {565., 642.} | 118.551 | 0.338958 | newly 48 weeks cumul | 280. | 623.18 | {578., 665.} | 122.564 | 0.346714 |
| 46 | newly 3 weeks spec | 223. | 478.77 | {349., 556.} | 114.695 | 0.325155 | newly 3 weeks cumul | 1040. | 2268.93 | {1708., 2558.} | 118.166 | 0.332361 | newly 5 weeks spec | 61. | 215.07 | {160., 253.} | 252.574 | 0.540645 | newly 5 weeks cumul | 1223. | 2804.35 | {2113., 3143.} | 129.301 | 0.354222 | newly 48 weeks cumul | 1322. | 3229.26 | {2448., 3621.} | 144.271 | 0.38202 |
| 47 | newly 3 weeks spec | 132. | 142.72 | {124., 162.} | 10.803 | 0.0440123 | newly 3 weeks cumul | 614. | 807.66 | {771., 844.} | 31.5407 | 0.118796 | newly 5 weeks spec | 43. | 49.28 | {40., 59.} | 19.2558 | 0.0754454 | newly 5 weeks cumul | 740. | 942.02 | {893., 999.} | 27.3 | 0.104482 | newly 50 weeks cumul | 804. | 1010.93 | {958., 1069.} | 25.7376 | 0.0990514 |
| 48 | newly 3 weeks spec | 186. | 200.43 | {180., 222.} | 9.2957 | 0.0381021 | newly 3 weeks cumul | 969. | 1082.38 | {1051., 1114.} | 11.7007 | 0.0479646 | newly 5 weeks spec | 49. | 71.78 | {60., 85.} | 46.4898 | 0.162026 | newly 5 weeks cumul | 1115. | 1274.04 | {1240., 1308.} | 14.2637 | 0.0578111 | newly 50 weeks cumul | 1182. | 1381. | {1338., 1420.} | 16.8359 | 0.0674552 |
| 49 | newly 3 weeks spec | 8. | 5.56 | {2., 9.} | 39.5 | 0.266051 | newly 3 weeks cumul | 68. | 46.02 | {35., 57.} | 32.4412 | 0.178211 | newly 5 weeks spec | 3. | 1.45 | {0., 3.} | 60.3333 | 0.600468 | newly 5 weeks cumul | 72. | 50.13 | {38., 62.} | 30.5417 | 0.166965 | newly 52 weeks cumul | 76. | 51.16 | {40., 66.} | 32.7632 | 0.181491 |
| 50 | newly 3 weeks spec | 733. | 1089.1 | {1043., 1133.} | 48.5812 | 0.171777 | newly 3 weeks cumul | 2944. | 4657.02 | {4594., 4701.} | 58.1868 | 0.199154 | newly 5 weeks spec | 408. | 572.58 | {548., 596.} | 40.3382 | 0.146894 | newly 5 weeks cumul | 3889. | 6018.52 | {5962., 6068.} | 54.7575 | 0.189643 | newly 43 weeks cumul | 4985. | 7497.15 | {7448., 7559.} | 50.3942 | 0.177225 |
